# Brain Mitochondrial Dysfunction: A Possible Mechanism Links Early Life Anxiety to Alzheimer’s Disease in Later Life

**DOI:** 10.14336/AD.2022.0221

**Published:** 2022-07-11

**Authors:** Qixue Wang, Mengna Lu, Xinyu Zhu, Xinyi Gu, Ting Zhang, Chenyi Xia, Li Yang, Ying Xu, Mingmei Zhou

**Affiliations:** ^1^Institute for Interdisciplinary Medicine Sciences, Shanghai University of Traditional Chinese Medicine, Shanghai, China.; ^2^School of Pharmacy, Shanghai University of Traditional Chinese Medicine, Shanghai, China.; ^3^Department of Physiology, School of Basic Medicine, Shanghai University of Traditional Chinese Medicine, Shanghai, China.; ^4^Shanghai Frontiers Science Center of TCM Chemical Biology, Institute of Interdisciplinary Integrative Medicine Research, Shanghai University of Traditional Chinese Medicine, Shanghai, China

**Keywords:** Alzheimer’s disease, anxiety, early life, mitochondrial dysfunction, oxidative stress

## Abstract

Alzheimer’s disease (AD) is usually manifested in patients with dementia, accompanied by anxiety and other mental symptoms. Emerging evidence from humans indicates that people who suffer from anxiety in their early life are more likely to develop AD in later life. Mitochondria, the prominent organelles of energy production in the brain, have crucial physiological significance for the brain, requiring considerable energy to maintain its normal physiological activities. Net reactive oxygen species (ROS) was produced by mitochondrial impairment, in which oxidative stress is also included, and the production of ROS is mostly more than that of removal. In this paper, we propose that as a critical process in brain pathology, mitochondrial dysfunction caused by anxiety triggering oxidative stress might be a possible mechanism that links early life anxiety to AD in later life. Several pivotal physiological roles of mitochondria are reviewed, including functions regulating glucose homeostasis, which may disrupt in oxidative stress. Increased levels of oxidative stress are constantly shown in anxiety disorder patients, and antioxidant drugs have promise in treating anxiety. In the early stages of AD, mitochondrial dysfunction is concentrated around senile plaques, a landmark lesion composed of aggregated Aβ and Tau protein. In turn, the accumulated Aβ and Tau disrupts mitochondrial activity, and the tricky physiological processes of mitochondria might be significant to the course of AD. In the end, we conclude that mitochondria might present as one of the novel therapeutic targets to block oxidative stress in patients with anxiety disorders to prevent AD in the early stage.

## 1. Introduction

Alzheimer’s disease (AD) is the most common form of dementia in later life. The pathophysiological mechanism of AD progression to dementia is extracellular neuroinflammatory plaque and fiber accumulation (β amyloid aggregation, neuronal fiber tangles and hyperphosphorylated tau protein aggregation, etc.), leading to extensive neuronal loss and altered neurotransmitter systems. β amyloid (Aβ) plaque-like deposition results from incomplete clearance or overproduction of peptides and is the most critical aspect of AD pathogenesis, which further leads to neurofibrillary tangles and cell death, culminating in cognitive dysfunction [1]. Recently, growing evidence shows that early life anxiety has enduring effects on the central nervous system which has matured and, consequently, on behaviors in later life [2-6]. One of the meta-analyses on the association between anxiety and risk of AD indicated that anxiety is slightly involved with an increased risk of AD based on a total of 24,528 participants, yet a high degree of heterogeneity across the studies and the inconsistency in the way of anxiety measuring might lead to the differences to be insignificant [7]. Another recent meta-analysis confirmed that anxiety significantly increases the risk of dementia by 29% [8]. Mitochondrion-centered hypometabolism is a key feature of brain aging and AD [9]. Aging is a process characterized by the gradual loss of tissue and organ function. The oxidative stress theory of aging is based on the hypothesis that age-associated functional losses are due to the accumulation of reactive oxygen species (ROS) -induced damages [10]. Given the long-term association between anxiety disorder and dementia, brain mitochondrial dysfunction and oxidative stress have a causal link between anxiety and AD.

Compared to other organs, the brain is susceptible to oxidative stress. Considering the brain not only has a high rate of oxygen consumption, but also has high requirements for transition metal ions and antioxidant enzymes that are involved in redox reactions [11]. There is growing evidence that free radical oxygen damage, mitochondrial dysfunction, accumulation of oxidative condensates, inflammation and defects in scavenging proteins constitute a complex system of pathological damage that act together on brain nerve cells, a pathological mechanism that has similarities in neurodegenerative diseases (e.g., AD, Parkinson’s disease). On the other hand, the differences of the pathology of neurodegenerative diseases are recognized as the result of complex interactions among genetic, environmental, aging, lifestyle and psychosocial factors. AD is the most common neurodegenerative disease, accounting for an estimated 60-70% of all dementia cases worldwide [12]. Therefore, here we attempt to elaborate the role played by oxidative stress in the development of AD in later life in patients with early life anxiety. A study has shown that oxidative stress plays a significant part in the progression of AD [13]. Mitochondrial and oxidative damage will cause an unbalance both in the production and removal the ROS, thereby increasing the net ROS [14]. As we know, the energy supply of neurons entirely relies on mitochondrial oxidative phosphorylation (OXPHOS). When OXPHOS is impaired, neurons have limited ability to obtain energy through glycolysis, which makes them particularly vulnerable to mitochondrial dysfunction [15]. Excessive stimulation of the electron transport chain can promote the overproduction of mitochondrial reactive oxygen species, leading to oxidative stress. Many disease mechanisms involve oxidative stress. Mitochondrial proteins, lipids, and DNA will undergo various oxidation reactions, and nerve cells will also be impaired [16]. Several studies pointed anxiety might be associated with decreased antioxidant defense capabilities and up-regulated oxidative damage to proteins, lipids, and nucleic acids. Notably, the oxidative modification of proteins is considered a potential factor in the occurrence and progression of anxiety [17].

In the following sections, we summarized several mechanisms that may account for this connection, focusing on brain mitochondrial dysfunction, supposed to be possible mechanisms that link early life anxiety to AD in later life.

## 2. Early life anxiety proved a risk factor for AD clinically

The prevalence of anxiety symptoms in AD is about 40%, and it can be a prelude to AD. Anxiety often occurs early in the course of AD, especially among patients with mild cognitive impairment (MCI), mild dementia, or early-onset forms of the disease, and can promote progression and conversion from MCI to dementia (Table 1) [18].

Although there is a consensus on the positive association between dementia and anxiety, it is noted that results vary considerably from study to study, with a higher probability of dementia of up to 46.7% reported recently [19]. The presence of cognitive impairment, reduced ability to perceive symptoms and verbal communication disorders can make the diagnosis of elderly patients more complex [20]. Anxiety in AD patients tends to present differently from anxiety in non-demented elderly, which differs from that of younger patients [21]. This discrepancy is due to the different thresholds set by the diagnostic criteria [7,22]. In the third edition of the Diagnostic and Statistical Manual of Mental Disorders (DSM-III), anxiety neurosis was split into generalized anxiety disorder (GAD) and panic disorder. Since the revision of DSM-III, worries about various life events have been gradually emphasized as the distinctive symptom of GAD. Thus, the cognitive aspect of anxiety has become a core criterion for GAD [23]. 41.73% of the registered psychiatric inpatients were diagnosed with dementia in which AD was the dominant subtype [24]. The prevalence of anxiety disorder is also disproportionally high among patients diagnosed with MCI, which presents as a relatively mild form of the cognitive deficit between normal aging and dementia [25]. It is found that neuropsychiatric symptoms can be risk factors for cognitive decline in clinically normal older adults [26]. The reported prevalence of anxiety in MCI patients ranged between 9.9%-52% [27]. Even before MCI or other cognitive symptoms, anxiety disorder appears to predict the progression of AD [28]. Gulpers et al. found that anxiety is associated with an increased risk for cognitive impairment and dementia in the community. Stronger associations were driven by higher age, suggesting a prodromal symptom [29]. Other studies, however, indicate that anxiety disorder is not a risk factor. Still, an early symptom of the neurodegenerative process of AD, advocating an overlap between the neuropathological events causing cognitive deficits and those causing anxiety disorder symptoms in dementia [30,31]. A large body of structural and functional imaging data show that a neuropathological basis in cognition correlates with the progression of anxiety disorder.

**Table 1:** Anxiety as a risk factor of Alzheimer’s disease in clinical trials.

Year/Study design/Group	Mean age (SD)	Country/Source of data/Study setting	Diagnostic criteria: Anxiety/AD	Main findings	Conclusions	Ref.
**1999/None/Community-dwelling AD patients (n=523)**	\	USA/University of Washington and Group Health Cooperative of Puget Sound/Clinic	DSM-IV/CT	Anxiety symptoms were common, occurring in 70% of subjects.	Anxiety symptoms were common and significantly related to ADL and additional neuropsychiatric problems in this sample.	[149]
**2003/Cross-sectional study/Probable AD (n=115), VaD (n=43), FTD (n=33), Controls(n=40)**	77.2(7.6); 75.1(9.8); 65.8(8.5); 73.6(6.1)	USA/UCLA Alzheimer’s Disease Center database/Clinic	NPI/NINCDS-ADRDA	In AD, anxiety was more prevalent among patients with a younger age at onset (under age 65).	In AD, anxiety is common in those with more severe cognitive deterioration and an earlier age at onset.	[150]
**2010/None/a-MCI (n=19), Ade (n=15), Adm (n=12), HS (n=23)**	73.3(6.9), 75.5(7.0), 70.0(8.2), 63.9(9.5)	Italy/Pecialist dementia clinic of Santa Lucia Foundation/Clinic	NPI-12, VBM/MRI	Anxiety was present in both a-MCI and AD.	Anxiety is present since the earliest AD stages.	[151]
**2013/Prospective pilot study/Early stage of AD patients (n=54), Healthy controls (n= 64)**	76.9(8.5); 69.3(8.7)	Switzerland/Memory Clinic of the Old Age Psychiatry Service of the Lausanne University /Clinic	NPI-Q/NINCDS-ADRDA	Behavioral and psychological symptoms, in particular apathy, anxiety, are frequent occurrences in early-stage AD.	Premorbid personality was not associated with BPS in early stage of AD, although complex and non-linear relationships between the two are not excluded.	[152]
**2014/None/EOAD patients (n=23), LOAD patients (n=22)**	57.68(4.19); 80.32(5.89)	USA/Departments of Neurology and Geriatric Psychiatry at the Veterans Affairs Greater Los Angeles Healthcare Center /Clinic	NPI/-	EOAD patients had significantly more anxiety symptoms than LOAD patients.	Among LOAD patients, anxiety was associated with psychotic and activating psychiatric symptoms.	[153]
**2015/Prospective cohort study/Healthy, older adults (n=333)**	70.0(6.8)	Australia/Australian Imaging, Biomarkers, and Lifestyle Study/Clinic	HADS/PET, APOE genotyping	A positive Aβ status at baseline was associated with elevated anxiety symptoms; Compared with the Aβ+, low-anxiety group, slopes of cognitive decline were significantly more pronounced in the Aβ^+^ high-anxiety group.	Elevated anxiety symptoms moderate the effect of Aβ on cognitive decline in preclinical AD, resulting in more rapid decline in several cognitive domains.	[154]
**2015/Cross-sectional study/Mild Dementia (n = 55), Moderate Dementia (n = 17), Severe Dementia (n = 20)**	58.8(4.1); 58.8(3.7); 59.7(3.1)	Japan/Kumamoto University Hospital/Clinic	NPI/MRI	Scores of the anxiety increased significantly with increased dementia severity.	Hallucinations, depression, and anxiety showed different patterns in EOAD.	[155]
**2017/None /EOAD (n = 16), NCs (n = 19)**	57.6 (4.2); 55.9(8.9)	USA/Greater Los Angeles Healthcare Center/Clinic	NPI/-	On the Neuropsychiatric Inventory, the ORs among the EOAD patients significantly correlated with anxiety scores.	Anxiety in mild EOAD may be associated with widening attentional refocusing to socioemotional stimuli, possibly reflecting decreased sensorimotor gating in the entorhinal cortex.	[156]
**2018/Longitudinal study/Community-dwelling, cognitively normal elderly individuals (n=270)**	73.6(6.1)	USA/Harvard Aging Brain Study/Community sample	Anxiety-concentration cluster/PiB-PET, Hollingshead score, AMNART	Higher PiB binding also predicted steeper rates of increase for anxiety-concentration scores.	A direct or indirect association of elevated amyloid beta levels with worsening anxious-depressive symptoms and support the hypothesis that emerging neuropsychiatric symptoms represent an early manifestation of preclinical Alzheimer's disease.	[157]
**2019/Longitudinal study/No AD (n=3968), Incident AD(n=87)**	72.83(9.03); 83.72(7.13)	Spain/Zaragoza Dementia and Depression project/Population-based	GMS-AGECAT/-	Significant association between anxiety cases at baseline and AD risk in the univariate analysis that persisted in the fully adjusted model; No significant association between 'subcases' of anxiety at baseline and AD risk was found.	Late-life, clinically significant anxiety (but not subclinical anxiety) seems to increase the risk of AD, independently of the effect of several confounders, including depression.	[158]
**2019/None/EOAD(n=24), LOAD(n=56)**	59.3(6.0), 82.3(4.9)	UK/Memory services of the South London and Maudsley NHS Foundation Trust /Clinic	NPI/-	Participants with EOAD were significantly worse on anxiety subscales.	The NPS severity was similar between EOAD and LOAD although EOAD had higher symptom prevalence and career distress.	[159]
**2020/Longitudinal study/CU(n=104), MCI(n=53)**	52(50.0); 22(41.5)	Sweden/Swedish BioFINDER study/Clinic	HADS/MRI, Amyloid PET scanning	Apathy and anxiety were shown related to Aβ deposition and predicted cognitive decline; Anxiety also interacted with amyloid status to predict faster cognitive deterioration.	The associations between apathy and anxiety with Aβ deposition and cognitive decline point to these symptoms as early clinical manifestations of Alzheimer's disease.	[160]

DSM-IV: the diagnostic and statistical manual of mental disorders, 4th ed; CT: computed tomography; ADL: activities of daily living; VaD: vascular dementia; FTD: frontotemporal dementia; NPI: neuropsychiatric inventory; NINCDS-ADRDA: The National Institute of Neurological and Communicative Disorders and Stroke and the Alzheimer's Disease and Related Disorders Association; a-MCI: amnestic mild cognitive impairment; Ade: early Alzheimer’s disease; Adm: moderate Alzheimer’s disease; VBM: voxel-based-morphometry; MRI: Magnetic resonance imaging; NPI-Q: neuropsychiatric inventory questionnaire; BPS: behavioral and psychological symptoms; EOAD: early-onset Alzheimer disease; LOAD: late-onset Alzheimer disease; HADS: hospital anxiety and depression scale; PET: positron emission tomography; AMNART: American national adult reading test; GMS-AGECAT: Geriatric mental state schedule- automated geriatric examination for computer assisted taxonomy.

The advent of cerebrospinal fluid (CSF) and fluorodeoxyglucose-positron emission tomography (FDG-PET) methods for the assessment of amyloid and tau has allowed the characterization of these more direct AD biomarkers to identify early stages and high-risk populations [32]. In the case of amyloid, its rise in the brain is thought to precede structural magnetic resonance imaging and FDG-PET changes [33]. Therefore, several studies have attempted to determine the relationship between anxiety and these in vivo measures of AD pathology (Table 1). Overall, anxiety has been strongly associated with direct biomarkers of amyloid and tau in vivo, in the same way as FDG-PET abnormalities. Furthermore, as with hypometabolism on FDG-PET, there is evidence that anxiety appears to enhance the effect of brain amyloid to progression, although its role in normal subjects seems less clear. The regional distribution of amyloid affects anxiety also needs to be further explored, as it seems to vary based on stage. Such work would also benefit from the advent of specialized tau PET imaging, as regional tau distribution has been shown to correlate better with symptoms than amyloid plaques [34].

## 3. Mitochondrial function in the physiological and pathological brain

There are roughly two sources of mitochondria in the brain. One is called non-synaptic mitochondria, mainly from neurons and glial cell bodies, and the other is called synaptic mitochondria, mainly from nerve terminals [35]. Using two-dimensional differential gel electrophoresis and mass spectrometry to detect synaptic proteins, the data show significant differences in superoxide dismutase [Mn] (Sod2) and complement component 1Q subcomponent binding protein (C1qbp), which supports the idea that synapses are highly sensitive to oxidative stress[36]. ROSs are small biological molecules, including superoxide (O^2-^), hydrogen peroxide (H_2_O_2_), and hydroxyl (OH^-^) free radicals, which are continuously and naturally produced in aerobic organisms [37]. The role of mitochondria as an organelle of energy metabolism is more prominent. Compared with nonneuronal cells, oxidative damage caused by mitochondria had a greater influence on the central neuron [38]. It is found that mitochondria provide energy for messaging by focusing on presynaptic terminals through active transport. Mitochondria target metabolic fluxes to generate power by interacting with glial cells around the synapse [36]. Most of the brain's energy supply is consumed by neuronal postsynaptic currents. It is essential to describe how neurons control the mitochondria where it distributes or displays the understanding of how synapses communicate with each other [39].

The neuronal activity requires the mitochondrial OXPHOS system at all times. Respiratory complexes I to IV and complex V form the electron transport chain (ETC). The coordinated operation of the ETC makes the mitochondria release energy usually. These redox reactions generate energy in the form of ATP, and release ROS, which can participate in cell signal transduction and ultimately cause oxidative stress [40]. Metabolic coordination between neurons and astrocytes is critical for the brain’s health [41]. In the brain, mROS derived from astrocytes are produced at an order of magnitude faster than neurons [42]. When the mitochondrial membrane-associated type 1 cannabinoid receptor (mtCB1) is activated by cannabinoids, phosphorylation of NDUFS4, a subunit of mitochondrial complex I, is reduced, further leading to instability and low activity of complex I. As a result, the ROS generated by astrocytes occurs to a reduction, which impacted the lactate levels produced by the glycolytic process through the hypoxia-inducible factor 1 pathway. All these interactions would act on neuronal redox stress [43]. Thus, when the stability of the mitochondrial complex is disrupted, the mROS initially released by astrocytes is transmitted to the neurons, thus subjecting the neurovascular unit to an energetic crisis and ultimately leading to neurodegeneration (Table 2) [44].

**Table 2:** Mitochondrial dysfunctions in AD.

Experiment models	Mitochondria dysfunctions	Mechanisms	Ref.
**ApoE KO mice; Aβ1-40**	Oxidative stress, mitochondrial dysfunction and caspase activation are up-regulated.	Thiobarbituric acid-reactive substances were at a higher level, which is in accordance with the situation of ApoE KO mice synapses occurred to lipid peroxidation.	[161]
**Tg2576 mice**	Mitochondrial stress response; altered the respiratory chain complexes I and III's protein subunit; down-regulated the state 3 respiration and noncontinuous brain mitochondria respiration; reduced glucose metabolism.	The changes in mitochondrial proteome and function in Tg2576 mice brain precede plaque pathology.	[162]
**Tg mice**	Synaptic mitochondria accumulate the Aβ; mitochondrial alterations; up-regulate the transform of mitochondrial permeability up-regulate the mitochondrial oxidative stress; down-regulate the ECT function and the COX activity;	Synaptic mitochondria rich in Aβ would rather have Aβ-induced damage, and synaptic mitochondrial dysfunction is linked with the development of synaptic degeneration in AD.	[91]
**Overexpressing ABAD Tg mice**	Neuronal oxidative stress damage and memory loss.	The active site used to inhibit NAD binding has a substantial deformation shown in the crystal structure of Aβ-bound ABAD. ABAD peptide specifically prevents ABAD-Aβ from interaction and further restraints apoptosis triggered by Aβ and neuron free-radical generation.	[163]
**CypD-deficient mAPP mice**	Cortical mitochondria lack of CypD leads to having immunity in mitochondrial swelling and permeability transition induced by Aβ and Ca^2+^.	The synaptic function can be improved by CypD deficiency.	[164]
**CypD-deficient mAPP mice**	Down-regulated calcium caused by mitochondrial swelling; up-regulated the uptake ability of mitochondrial calcium; improved mitochondrial respiratory function.	Neuronal and synaptic stress that CypD mediated mPTP can be triggered by mAPP and oxidative stress.	[165]
**CypD-deficient mice (primary cortical neurons and astrocytes)**	The reason for synaptic versus nonsynaptic mitochondria has a difference in the Ca^2+^ handling is that in synaptic mitochondria, the levels of CypD are detected higher.	The neuronal mitochondria had a high level of CypD makes it vulnerable to mPTP.	[166]
**Tg mAPP/ABAD mice**	Spontaneous generation of hydrogen peroxide and superoxide anion, and decreased ATP, the release of cytochrome c from mitochondria and induction of caspase-3-like activity followed by DNA fragmentation and loss of cell viability.	ABAD-induced oxidant stress is related to cellular dysfunction accociated with AD.	[167]
**Hippocampal neurons from embryonic day 18 rats; Aβ35-25**	Impaired mitochondrial transport; morphological changes; inhibited mitochondrial transport by acting through GSK3β.	To determine the important acute effect of Aβ molecules on nerve cells, which may lead to various abnormalities of neuronal function under AD conditions.	[168]
**Human neuroblastoma M17 cells**	Abnormal mitochondrial distribution pattern; reduced mitochondrial density.	The overexpression of DLP1 may be through the process of repopulating neurons with mitochondria to prevent ADDL-induced synapse loss, indicating that abnormal mitochondrial dynamics are fragile for the synaptic abnormal induced by ADDL.	[169]
**Human neuroblastoma M17 cells (APP overexpression)**	The perinuclear area is surrounded by fragmented structure and abnormal distribution; upgraded ROS levels, decreased mitochondrial potential difference, and reduced ATP releasement.	Fragmented mitochondria and abnormal distribution account for the mitochondrial and neuronal loss.	[170]
**sAD patients’ fibroblasts**	The characteristic of abnormal mitochondrial distribution is that slender mitochondria accumulate in the pernuclear area of 19.3% of sporadic AD (sAD) fibroblasts; decreased DLP1.	The reason why the levels of DLP1 reduced and mitochondrial allocation is unusual is that up-regulated oxidative stress and amyloid production in AD cells.	[171]
**AD postmortem brain tissues, AβPP tg mice (primary hippocampal neurons)**	Abnormal mitochondrial dynamics increase as AD progresses.	Crucial factors, including the increased production of Aβ and the interaction of Aβ with Drp1, lead to mitochondrial fragmentation, abnormal mitochondrial dynamics and synaptic damage.	[172]
**Hippocampal neuron from C57BL/6day 1 pup; Aβ25-35 peptide**	Less level of motile mitochondria; less average speed of motile mitochondria; decreased mitochondrial length; less synaptic immunoreactivity.	In neurons of AD models, toxic Aβ can impair mitochondrial movement, shorten the length of mitochondria, and endanger synaptic loss.	[173]
**AD postmortem brain tissues; APP, APP/PS1 and 3XTg.AD mice**	Elevated mitochondrial fission-linked GTPase activity.	Aβ and phosphorylated tau and Drp1 are entangled with each other, causing damage to mitochondria and synapses, which in turn leads to cognitive memory deficits.	[92]
**AD postmortem brain tissues; CaMKIIα-tTA and tet-APPswe/ind mice**	Enhanced mitophagy; depletion of cytosolic Parkin; reduced anterograde and increased retrograde transport of axonal mitochondria.	Chronic mitochondrial stress associated with AD under pathophysiological conditions in vitro and in vivo effectively triggers Parkin-dependent mitochondrial autophagy.	[174]
**AD postmortem brain tissues; APP/PS1 mice**	Down-regulated mitophagy.	Mitophagy enhancement eliminates AD-related tau hyperphosphorylation in human neuronal cells and reverses the memory impairment of genetically modified tau nematodes and mice.	[86]

mPTP: mitochondrial permeability transition pore; ABAD: amyloid protein binding of alcohol dehydrogenase; COX: cytochrome c oxidase; GSK3β: glycogen synthase kinase 3β; DLP1: dynamin-like protein; OPA1: mitochondrial dynamin-like GTPase; Mfn1/2: mitofusin 1/2; ADDLs: amyloid-β-derived diffusible ligands; Opa1: mitochondrial dynamin-like GTPase 1; TOMM40: translocase of outer mitochondrial membrane 40.

## 4. Brain mitochondrial dysfunction in anxiety

The relationship between oxidative stress and anxiety symptoms was first described in 2001. In a non-clinical sample of healthy adults aimed at the levels of 8-hydroxy-2'-deoxyguanosine (8-OH-dG) in peripheral blood leukocytes, female subjects showed a positive relationship between the amount of 8-OH-dG and the psychosocial factors, such as anxiety [45]. In 2004, Sklan hypothesized that anxiety results from a complex, incompletely understood genome and external environment. According to his research, the acetyl-cholinesterase protein controls the termination of stress-enhanced acetylcholine signaling. In contrast, paraoxonase protein exhibits peroxidase-like activity, protecting blood proteins from oxidative stress. In addition, the instantaneous score of state anxiety and the susceptibility score of trait anxiety seem to be related to enzyme activity [46]. Olsen et al. tested the water maze, the situational fearfulness experiment and the elevated maze in the overexpressing mitochondrial catalase mice and concluded that overexpression of catalase may be sufficient to improve cognitive performance and reduce anxiety in the absence of changes in oxidative levels [47]. In 2014, a series of behavioral tests carried out on transgenic mice expressing the COXIV zip code uncovered an “anxiety-like” behavioral phenotype, indicating that the axon transport of nuclear-encoded mitochondrial mRNAs plays a pivotal part in neuronal physiology and rodent behavior [48]. Several studies confirm this connection, contributing to a better understanding of the intimated interaction between brain mitochondria and anxiety [49].

### 4.1 Mitochondrial oxidative stress acts as a core of mitochondrial dysfunction, illustrating the mechanisms of anxiety

Some scholars have incorporated antioxidants with potential neuroprotective effects, such as ascorbic acid, into their treatment strategies when studying mental illness. In the face of mood and anxiety disorders that are currently difficult to treat, ascorbic acid supplements have been presumed to be candidate drugs because of their fast onset, low toxicity, and high tolerability treatment response [50]. There are two main types of drugs most commonly used in the treatment of recurrent anxiety disorder in the world, including selective serotonin reuptake inhibitors (SSRIs) and serotonin and norepinephrine reuptake inhibitors (SNRIs) [51]. The above preparations may have an underestimated antioxidant effect that could underly the potential mechanisms [52]. Thioredoxin (Trx) is an antioxidant protein that reverses the oxidation of protein cysteines and promotes the scavenging of reactive oxygen species. Incubation of hippocampal cells from HT22 mice with fluoxetine for five consecutive days revealed upregulation of Trx protein levels, followed by inhibition of protein sulphonation and nitrosylation, suggesting that fluoxetine may protect against oxidative stress[53]. The genetic fragility of mitochondria leads to a calcium imbalance and leads to hyperexcitability of serotonergic neurons, which is thought to be susceptible to oxidative stress [54]. Basu et al. used the three-photon microscope to image serotonin in living preimplantation mouse embryos. Live embryos pre-incubated with serotonin showed higher mitochondrial potential, indicating the potential to regulate mitochondria [55]. In a word, these observations suggest an exciting link between the regulation of mitochondrial oxidative stress by serotonin and the anxiolytic effects of SSRIs.

### 4.2 Brain mitochondrial regulation of glucose homeostasis

The hippocampus, amygdala, and prefrontal cortex are brain regions associated with memory and emotion. After repeated stressful stimuli, structural remodeling is induced, which decreases memory and increases anxiety-like behavior and aggression. Structural and functional magnetic resonance imaging studies of anxiety disorders have demonstrated that the human brain may be affected similarly [56]. Another coherent connection linked oxidative stress to anxiety is brain mitochondria regulation of glucose homeostasis. High anxiety can lead to the release of sympathetic hormones, thereby increasing cortisol and glucose levels, reducing the insulin release, or affecting the sensitivity and resistance of insulin hormones [57]. From September 2013 to May 2015, Zhou et al. conducted a study on 646 anxiety patients who met the revised standards of the 10th edition of the International Statistical Classification of Diseases and Related Health Problems. It is found that the general probability of abnormal blood glucose regulation in anxiety patients accounts for 24.61%. The results of this article partially confirm the great quantity of impaired glucose regulation in patients with anxiety. Impaired glucose in the young group is closely related to the hypothalamic-pituitary-adrenal axis. By contrast, glucose impairment in the elderly group is closely associated with changes in the hypothalamic-pituitary-thyroid axis [58]. Impaired glucose homeostasis can, in turn, affect the normal function of insulin. Early studies on insulin focused on the periphery, where insulin takes up glucose by transporting and activating glucose transporter proteins in cell membranes. Later it is found that the central nervous system can also take up glucose through insulin-mediated transduction. Therefore the brain insulin signaling process in anxious patients should also be noticed. The improved well-being and self-confidence in normal-weight individuals after eight weeks of intranasal insulin treatment suggests that brain insulin modulates mood-related neural signaling pathways [59]. Brain insulin receptors are widely distributed in the olfactory bulb, cerebral cortex, hippocampus, hypothalamus, amygdala and vomeronasal nucleus, and their number decreases with age [1,33,59,60]. Studies have shown that anxiety, fear and stress are correlated. This chronic stress can cause amygdala hyperactivity and damage to the prefrontal cortex (PFC) and hippocampal structures, thereby impairing emotion regulation [61].

### 4.3 Translocator protein 18 kDa (TSPO)

Prior to 1977, little was known about the transporter protein 18 kDa (TSPO), a protein-coding gene known as an alternative binding site for the benzodiazepine diazepam. It is an evolutionarily conserved, tryptophan-rich protein that, together with voltage-dependent anion channels and the adenine nucleotide transporter (ANT), forms the mitochondrial permeability transition pore (MPTP) [62]. Lower levels of TSPO are expressed in healthy brain tissue. Disorders associated with TSPO include hepatic encephalopathy and generalized anxiety disorder. In addition, TSPO is overexpressed in different types of neuroinflammatory diseases (e.g., AD) but at downregulated levels in psychiatric disorders (e.g., anxiety disorders). [63]. The finding that the rs6971 polymorphism of the TSPO gene leads to amino acid substitutions in the fifth transmembrane loop of human proteins also supports the anxiety-related disorder TSPO abnormality [64]. When administered a series of TSPO ligands, the rodent models showed relief of anxiety symptoms. Recent progress has focused on the clinical progression of TSPO drug ligands. For example, recent research has found that rodent models and human volunteers administered by the selective TSPO ligand XBD173 (AC-5216, Emapunil) have a better anxiolytic effect [65].

## 5. Mitochondrial oxidative stress and the dysfunction of cerebral mitochondria in AD

Globally, 47 million people have dementia, and this number is estimated to increase to 131 million by 2050. AD is the commonest cause of dementia, with approximately 5 million new cases occurring annually [66,67]. Advances in diagnostic methods, such as biomarkers and computer-aided diagnosis based on image analysis, have been a significant step forward [68,69]. The current "biological" conception of AD is based on consideration of three biomarkers: amyloid, tau, and "neurodegeneration" [70]. Aggregation of the misfolded protein, such as β-amyloid and tau, is always accompanied by oxidative stress [71]. A large number of studies have demonstrated the important role of mitochondrial dysfunction and oxidative damage in the pathogenesis of AD. Oxidative stress shows in the early stages of the AD brain, before the occurrence of Aβ and Tau deposition [72]. Recent literature uncovered by researchers revealed the relationship between oxidative stress and AD pathology. Oxidative stress may influence the process of APP or tau metabolism. For instance, when the c-Jun amino-terminal kinase and p38 mitogen-activated protein kinase (MAPK) became activated, β-secretase was released caused by oxidative stress [73]. Meikeqi et al. isolated 8-OH-dG, a marker of nucleic acid oxidation, from mitochondrial DNA in the cerebral cortex. Further, 8-OH-dG gradually increases with age and appears to be more in the AD brain, which supports the hypothesis of oxidative stress [74]. However, there is still doubt whether oxidative stress should be a cause or a consequence of AD [75,76]. Birnbaum et al. detected elevated ROS levels in postmortem brain samples and rodent models. They believe that the increase in ROS may act an indispensable part in the occurrence of sporadic AD before the appearance of amyloid and tau protein pathology [77]. Sivandzade et al. hypothesized that overproduction of ROS and electrophiles is one of the main precursors to the occurrence and progression of AD [78]. Mitochondria are an essential source of H_2_O_2_, and their dysfunction is usually related to changes in the redox state. Yin et al. showed that chronic oxidative stress is also a significant factor in the cognitive decline associated with AD [9].

### 5.1 Mitochondrial oxidative stress links brain ROS production to AD

In preclinical models or human patients, cigarette smoke/smoking is consistent with AD neuropathology. Smoking-related brain oxidative stress is a potential mechanism that promotes the pathological process of AD [79]. Emerging evidence suggests that sleep deprivation and circadian rhythm disorder may interact and increase the risk of AD progression by increasing local brain oxidative stress and reducing circulating melatonin levels [80]. People who suffer from Type 2 diabetes and obesity are easier developed to AD than usual. Owning the habit of a Mediterranean diet can alleviate the above situations and so does other activities, such as strengthening physical exercise. Mediterranean diet is rich in vitamins, polyphenolic compounds, and partial fatty acids, which can be absorbed into the body to reduce oxidative stress [81]. Insufficiency of cerebral perfusion and hypo-glycemia can cause neuroinflammation and oxidative stress, leading to brain synaptic dysfunction and neuronal degeneration/loss, leading to gray and white matter atrophy, cognitive dysfunction, and AD [82]. The central nervous system communicates with peripheral blood through cells and factors circulating to the brain, and vice versa. Microarray and metabolomics suggest that central and peripheral glycolytic abnormalities and dysfunction of oxidative pathways are similar [83]. The short-term or sustained destruction of the integrity of the blood-brain barrier (BBB) is related to decreased CNS protection and increased permeability of pro-inflammatory (such as cytokines, ROS) substances from peripheral blood into the brain [84]. Chronic neuroinflammation has been well studied so far, and it's reported that inflammatory cytokines released by highly sensitive microglia can further trigger oxidative stress, which could illustrate the pathology of AD [85].

### 5.2 Aβ deposition, Tau aggregation and brain mitochondria

Mitochondrial dysfunction is shown in brain tissues from clinical samples and AD transgenic rodent models [86]. Numbers of evidence have shown multiple defects in mitochondria at the early stage of AD, such as impaired mitochondrial bioenergetics impairment, damaged mitophagy, decreased mitochondrial biosynthesis, and abnormal mitochondrial oxidative stress [87-90]. Aβ aggregates at synaptic terminals and can also enter synaptic mitochondria to cause synaptic impairment and abnormal mitochondrial function. It is found that Aβ can bind to mitochondrial proteins, such as mitochondrial splitting protein Drp1, mitochondrial outer membrane protein VDAC, etc [91,92]. These abnormal effects cause excessive free radical production, enhance mitochondrial splitting and affect the mitochondria biogenesis, ultimately leading to mitochondrial dysfunction. Mitochondrial DNA is close to the site of oxidative reactions. It is highly susceptible to damage due to the absence of histones and repair mechanisms in mitochondrial DNA, and this oxidative stress damage is most evident at AD synapses [73]. Tau loses control of microtubules and neurons accumulate as neurofibrillary tangles [93]. Hyperphosphorylated and overexpressed tau proteins accumulate around mitochondria and cause abnormal mitochondrial distribution. Moreover, pathological tau takes part mitochondrial dynamics by regulating mitochondrial fission/fusion proteins. All that damage leads to neuronal loss and mitochondrial disorder [94]. The reduced/oxidized form of nicotinamide adenine dinucleotide plays an important role in redox homeostasis and energy requirements [95,96]. The first molecule that enters the ETC of the mitochondria, is nicotinamide dinucleotide. The ETC comprises four major protein-metal complexes (I-IV), which trigger ATP production since the electrons flow. ATP synthase, also termed complex V, which is linked with OXPHOS. When the electron flow through the ETC, Complex V comes to the synthesis of ATP [97]. Repairing the disabled mitochondria by supplementing nicotinamide adenine dinucleotide (NAD) has been confirmed as a promising strategy for the treatment of AD and other dementia [98].

## 6. Oxidative stresses triggered by mitochondria dysfunction is a possible mechanism that links early life anxiety to AD in later life

The source of ROS may mainly date back to brain mitochondrial dysfunction, which is seen as a therapeutic target in the CNS. In oxidative stress, mitochondrial dysfunction is of great importance, and now literatures on the pathogenesis of AD and anxiety disorder are mainly underlain on them. A large amount of literature, including clinical research and rodent model research, have determined that up-regulated releasement of oxidative stress is related to AD and anxiety.

### 6.1 Akt1

Akt is related to neurological diseases. It has three isoforms, Akt1/Akt2/Akt3, with specific brain cell types that may have different effects on behavior [99]. An essential aspect of the Akt1 function is cellular metabolism and energy production. In some cases, activation of Akt1 strongly increases oxidative stress and causes apoptosis when cells gradually accumulate excess free radicals [100]. Accordingly, ROS can be involved in the oxidative modification of Akt1, which causes synaptic dysfunction in AD. Therefore, there is a growing number of therapeutic strategies dedicated to promoting synaptic Akt1-mTOR signaling [101]. Arvanitakis et al. conducted a clinicopathological study, originating from a community-based cohort study. In the second analysis, normalized pT308 Akt1 was positively correlated with amyloid load and tau tangles density. In addition, normalized pT308 Akt1 is linked with lower levels of overall cognition [102]. Another report showed that Akt1-KO mice had down-regulated tau phosphorylation at the familiar site of microtubule affinity-regulated kinase 2 (PAR1/MARK2) [103]. William et al. used different ratios of glucose and insulin to culture human stromal vascular cells and differentiated adipocytes and found increased Aβ secretion in the conditioned medium. Adipocytes were cultured with Aβ leading to decreased insulin receptor substrate-2 and reduced Akt1 phosphorylation [104]. The associations between the 17-item Hamilton Depression Rating Scale, total score, four factors, and the Akt1 rs2494746 and rs3001371 polymorphisms were analyzed through UNPHASED software. It seemed that Akt1 polymorphisms are connected with anxiety symptoms in patients suffering from depressive disorder [105].

### 6.2 Insulin

Insulin acts on the brain to affect cognition and mood. Brain insulin resistance and reduced insulin sensitivity in the central nervous system are standard features of dementia and aging [106]. Patients with AD have been found clinically to exhibit hyperinsulinemia on both fasting glucose and oral glucose tolerance tests. However, chronic hyperinsulinemia decreases the number of insulin receptors and thus the amount of insulin entering the brain [107,108]. The researchers suggest that finding targets of insulin resistance in the central nervous pathway may be an essential aspect of improving cognitive impairment [109]. Specifically, activated insulin signaling had brain glucose homeostasis in control and was resistant to the damage caused by synaptic loss, (neuro)inflammation, and oxidative stress [110-113]. Astrocytes take control of the metabolic rate of brain glucose level as well as neuronal activity. The activation of insulin signaling by astrocytes in the hypothalamus is explained by the fact that astrocytes readily co-control glucose and circulate glucose metabolism in the CNS by regulating the transport of glucose into the BBB [114]. Revealing the role of the astrocyte GLP-1R pathway could study the integrity of mitochondria. Adaptive stress response caused by GLP-1R pathway disorder further mounts an increase of memory forming and systemic glucose moderation [115]. Intranasal and peripheral insulin administration can be turned into a potential therapeutic target for AD, showing that it can improve the memory of AD patients [110,116,117]. Furthermore, it is confirmed that brain insulin has a good effect on anxiolytic both in patients and rodent models. The critical pathological symptoms of AD, including impaired memory, inflammation markers, energy utilization of neuronal and brain activity, have been facilitated by insulin when carrying out the first clinical trials in MCI/AD patients [118]. A study of albino vom strain mice in an elevated maze test found that insulin at a dose of 1.0 IU/kg was found to exert anxiolytic effects [119]. Soto and co-workers knocked out insulin receptors and IGF-1 receptors in the hippocampus or central amygdala of mice, which in turn led to defective insulin signaling, as evidenced by decreased levels of GluA1 subunits of the glutamate AMPA receptor on the one hand and increased levels of anxiety-like behavior, cognitive impairment, and metabolic disturbances (e.g., glucose intolerance) on the other. [120].

### 6.3 Serotonin and brain glucose homeostasis

The search for therapeutic targets to treat anxiety through the angiotensin-converting enzyme [ACE] type 2 (ACE2), angiotensin [Ang]-(1-7), and Mas receptor pathways of the renin-angiotensin system is promising [121]. SSRIs and SNRIs are first-line drugs to treat anxiety disorders [122]. The serotonin system is of great importance to anxiety pharmacology and molecular imaging [123]. Dopa decarboxylase is a crucial enzyme that participates in synthesizing both dopamine and serotonin [124]. It has been established that excitation mediated by dopaminergic (DA) neurons in the ventral tegmental area (VTA) and the mesocortical border pathway is associated with the progression of anxiety. Current research indicates that the dopamine D2 receptor signaling pathway that connects the VTA to the basolateral amygdala regulates fear and anxiety [123,125]. The dorsal medial part of the dorsal raphe nucleus is preferentially innervated by important forebrain structures related to anxiety state regulation, which can project the distributed nervous system that regulates anxiety. And partial serotonergic neurons in this brain area are activated by anxiety stimulation [126]. More and more importance is attached to the relationship between anxiety plots and other alterations, including changed γ-aminobutyric acid levels [127], increased insulin secretion and insulin resistance [128], and oxidative stress [129-131], which provides a promising target for anxiolytics. An extensive study on the serotonergic system has revealed a possible target for treating anxiety disorders considering its relationship with memory and oxidative stress, including behavioral/psychological symptoms of AD and dementia [132-134]. Furthermore, one of several mechanisms by which serotonin may directly affect the pathogenesis of AD is to up-regulate the α-secretase activity through 5-hydroxytryptamine (HT)4 receptors [135]. Ma et al. showed that fluoxetine effectively attenuated tau hyperphosphorylation at Ser396 [136].

Serotonin, also termed 5-hydroxytryptamine (5-HT), is of great importance in neurotransmitters, growth factors, and hormones, which account for a series of physiological functions [137]. Another way that 5-HT is involved with anxiety disorder and AD is to modulate brain glucose metabolism. There is some evidence that SSRIs have a beneficial effect on glucose homeostasis [138]. In addition, fluoxetine has been shown to reverse apoptosis and mitochondrial dysfunction by modulating mitochondrial respiratory chain components and Krebs cycle enzymes, and to affect mitochondria-related redox parameters [139]. Hydrogen sulfide targets modulate oxidative stress and neuroplasticity to treat pathological anxiety [129]. Researchers believe that the anxiety-related brain area involves the dorsal raphe nucleus. The serotonergic neurons in the dorsal/tail part can project to the limbic region of the forebrain [140]. The gut microbiome is also an effective medium for synthesizing intestinal-derived serotonin. The peripheral source of serotonin is itself a regulator of glucose homeostasis [141]. Knockout of 5-HT2CR on pro-opiomelanocortin neurons in hypothalamus arcuate nucleus (ARC) of mice did not affect body weight but showed hyperglycemia, hyperinsulinemia and insulin resistance, suggesting a clear role of central serotonin signaling in the regulation of glucose metabolism [142]. The clinical efficacy of fluoxetine plays a new mechanism through fluoxetine targeting glucose metabolism by regulating the palmitoylation of the glucose transporter [143]. To figure out how the shell of the nucleus accumbens (sNAc) serotonin affects systemic glucose metabolism, Deepenbroek et al. inserted microdialysis in the bilateral sNAc of male Wistar rats. One hour later, they detected upregulated blood glucose concentrations but no change in glucoregulatory hormones in the sNAc. [144]. It is worth noting that some experiments have addressed the effectiveness of using SSRIs to promote 5-HT-mediated synaptic communication to control anxiety in patients with dementia [145]. Other trials targeting SSRIs seem to be necessary to earn their potential value in treating AD (Table 3) [146].

**Table 3:** Current studies on pharmacological treatment of agitation and psychosis in AD with anxiolytics.

Mechanism of action	Name	Study population	Treatment	Results/status of the study	Study ID (ClinicalTrial.gov)
**A selective 5-hydroxytryptamine (HT)2A receptor inverse agonist/antagonist**	Pimavanserin	AD psychosis	Pimavanserin 34 mg vs. PLC	Significant improvement for pimavanserin Primary endpoint (week 6): Mean change in the Neuropsychiatric Inventory-Nursing Home version psychosis score; Pimavanserin versus PLC: -3.76 points (SE = 0.65) vs -1.93 points (0.63) (mean difference -1.84 [95% CI -3.64 to -0.04], Cohen’s d = -0.32; p = 0.045); No significant advantage for pimavanserin vs PLC at week 12 (treatment difference -0.51 [95% CI -2.23 to 1.21]; p = 0.561);	ACP-103-019
		AD psychosis	Pimavanserin 34 mg vs. PLC	Significant efficacy in patients with higher baseline severity of psychotic symptoms (delta = -4.43, Cohen’s d = -0.73, p = 0.011); Pimavanserin vs PLC: ≥30% improvement was 88.9% vs. 43.3% (p < 0.001); ≥50% improvement was 77.8% vs. 43.3% (p = 0.008);	ACP-103-019
**A potent 5-HT2A antagonist, an inhibitor of serotonin reuptake**	Lumateperone (ITI-007)	Agitation in patients with dementia, including AD	A phase 3, 4-week, randomized, double-blind, placebo-controlled, multi-center study (ITI-007, 9 mg/d vs. PLC)	No results posted	NCT02817906
**Serotonin and norepinephrine reuptake inhibitor**	Dextromethorphan	Agitation in patients with AD	A phase 3, 12-week, multicenter, randomized, double-blind, placebo-controlled, parallel-design study (AVP-786 (dose 1) vs AVP-786 (dose 2) vs. PLC)	Ongoing (study is recruiting participants)	NCT03393520
**Selective serotonin reuptake inhibitor**	Escitalopram	Agitation in AD	A phase 3, 12-week, randomized, double-blind, placebo-controlled trial (5-15 mg/day (target: 15mg/day if tolerated) of escitalopram vs. PLC)	Ongoing (study is recruiting participants)	NCT03108846
**A partial 5-HT1A receptor agonist**	Tandospirone	AD psychosis	A phase 4, 12-week, randomized, open-label, parallel-group study (Tandospirone, 30-60 mg/d + Donepezil, 10 mg/d vs Donepezil, 10 mg/d.)	No results posted	NCT03151382
**Serotonin receptor antagonists and reuptake inhibitors**	Trazodone	AD psychosis	A phase 3, 14-days, randomized, double-blind, placebo-controlled study (Trazodone tablets, 50 mg/d vs. PLC)	No results posted	NCT01142258
**An analog of the inhibitory neurotransmitter gamma-aminobutyric acid (GABA)**	Gabapentin Enacarbil	Agitation in AD	A phase 4, pilot, 8-week, double-blind, placebo-controlled randomized clinical trial (GEn, 300 mg/d vs. PLC.)	Ongoing (study is recruiting participants)	NCT03082755

data available at: ClinicalTrials.gov (accessed February 12, 2022); filters used: agitation, psychosis, anxiolytics, and AD; Studies: recruiting; not yet recruiting; active, not recruiting; enrolling by invitation; PLC, placebo; AD, Alzheimer’s disease; GEn, Gabapentin Enacarbil.

## 7. Conclusion and prospect

**Figure 1.**
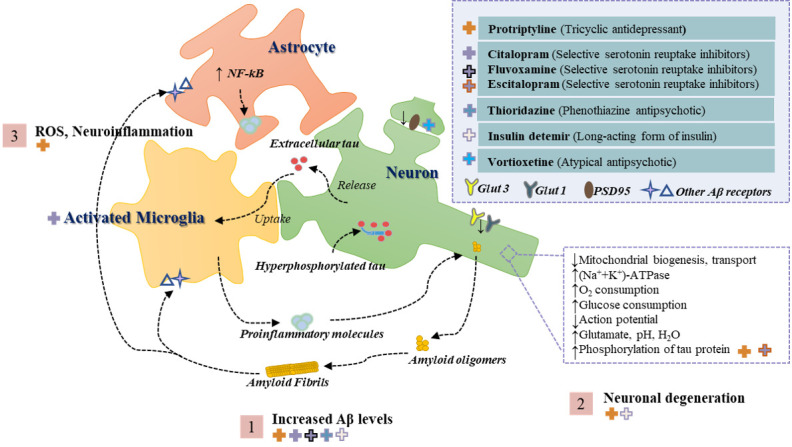
**Overview of the pathogenesis of AD triggered by extracellular and intravascular Aβ deposition**. Step 1 shows the interaction of Aβ oligomers and fibronectin with neuronal cells through several receptors which in turn triggers an overall increase in brain Aβ levels. Step 2 shows that neurons suffer from impaired mitochondrial bioenergetics and glucose homeostasis and thus degeneration. Step 3 illustrates that the neuroinflammatory environment and oxidative stress to which the neuronal cells are exposed can exacerbate the pathological process of AD. In addition, the top right corner of the figure shows the anxiolytic drugs reported in the literature for the treatment of AD models, with the crosses on the left corresponding to the respective antipathogenic mechanism in the figure and the classification of the drug in parentheses. Abbreviations: Aβ, amyloid peptide; GLUT, glucose transporter; NF-κB, nuclear factor kappa light chain enhancer of activated B cells; ROS, reactive oxygen species; PSD95, postsynaptic density protein-95.

As we all know, throughout the life cycle, early exposure to anxiety controls lifelong sensitivity to subsequent stress. Animals susceptible to anxiety disorders exhibit behaviors related to cognitive impairment. Although mitochondria have electron transport chains capable of producing ROS, the network of antioxidant defense systems is also developed [73]. Mitochondrial oxidative stress is caused by an imbalance between ROS generation and ROS detoxification. The observation that enhanced mitochondrial antioxidant defense reduces anxiety symptoms supports the importance of net mitochondrial ROS in producing anxiety. Discover the key molecular mechanisms that mediate the effects of anxiety in AD from mitochondrial dysfunction and make feasible biological targets that used to treat anxiety-induced sensitivity in early life, which may be the control of potential AD-related neurodegeneration diseases of this pharmacological target on possible clinical research has opened the door (Fig. 1).

The lifetime prevalence of anxiety is a known relatively decisive risk factor for AD, and it is reflected in many behavioral models of AD. Previous studies have shown that anxiety in the early stages of life can lead to a folding increase in the risk of adult AD, depending on its timing, intensity, and specific characteristics. It is also known that anxiety in the early stages of life will increase behavioral sensitivity, making people more affected by anxiety in the later stages of life. It will have a particularly strong effect on the nucleus accumbens (an important component of the brain's reward system). Through two functional magnetic resonance imaging studies, Li et al. found that anxiety affects processing efficiency far greater than performance efficiency, and the effect on processing efficiency of brain areas related to spatial working memory is much greater than that of verbal working memory, and the central brain area involved in the medial prefrontal lobe and anterior cuneiform lobe. At the same time, the activities of the medial frontal and subparietal lobules related to working memory of individuals with high trait anxiety are enhanced, the connection between the insula and the cingulate gyrus related to self-awareness is enhanced, but the connection between the cingulate gyrus and the amygdala responsible for emotion regulation is weakened. This part of the results reveals the unique emotions of anxious individuals and the brain networks related to working memory. In short, these results suggest an important cognitive basis for anxiety disorders and provide unique evidence for the neural mechanism of the interaction between anxiety and working memory [147,148].

In the future, it will be important to understand how mitochondrial changes in the neural circuits of anxiety and working memory. The importance of determining the characterization of mitochondrial function in different disease processions is much more than that only to consider the progress of research methods on mitochondria. Of note, anxiety phenotype shaped by brain mitochondrial dysfunction could contribute to the outcome of mitochondrial biogenesis, mitochondrial dynamics. Attaching more importance to the way how mitochondria shape the early life anxiety phenotypes that further leads to AD holds great promise among the efforts in the early prevention of AD of the future.
